# Novel Peptide Inhibitors for Lactate Dehydrogenase A (LDHA): A Survey to Inhibit LDHA Activity via Disruption of Protein-Protein Interaction

**DOI:** 10.1038/s41598-019-38854-7

**Published:** 2019-03-18

**Authors:** Farzaneh Jafary, Mohamad Reza Ganjalikhany, Ali Moradi, Mahdie Hemati, Sepideh Jafari

**Affiliations:** 1 Department of Clinical Biochemistry, Faculty of Medicine, Shahid Sadoughi University of Medical Sciences, Yazd, Iran; 2 Department of Biology, Faculty of Sciences, University of Isfahan, Isfahan, Iran

## Abstract

Lactate dehydrogenase A (LDHA) is a critical metabolic enzyme belonging to a family of 2-hydroxy acid oxidoreductases that plays a key role in anaerobic metabolism in the cells. In hypoxia condition, the overexpression of LDHA shifts the metabolic pathway of ATP synthesis from oxidative phosphorylation to aerobic glycolysis and the hypoxia condition is a common phenomenon occurred in the microenvironment of tumor cells; therefore, the inhibition of LDHA is considered to be an excellent strategy for cancer therapy. In this study, we employed *in silico* methods to design inhibitory peptides for lactate dehydrogenase through the disturbance in tetramerization of the enzyme. Using peptide as an anti-cancer agent is a novel approach for cancer therapy possessing some advantages with respect to the chemotherapeutic drugs such as low toxicity, ease of synthesis, and high target specificity. So peptides can act as appropriate enzyme inhibitor in parallel to chemical compounds. In this study, several computational techniques such as molecular dynamics (MD) simulation, docking and MM-PBSA calculation have been employed to investigate the structural characteristics of the monomer, dimer, and tetramer forms of the enzyme. Analysis of MD simulation and protein-protein interaction showed that the N-terminal arms of each subunit have an important role in enzyme tetramerization to establish active form of the enzyme. Hence, N-terminal arm can be used as a template for peptide design. Then, peptides were designed and evaluated to obtain best binders based on the affinity and physicochemical properties. Finally, the inhibitory effect of the peptides on subunit association was measured by dynamic light scattering (DLS) technique. Our results showed that the designed peptides which mimic the N-terminal arm of the enzyme can successfully target the C-terminal domain and interrupt the bona fide form of the enzyme subunits. The result of this study makes a new avenue to disrupt the assembly process and thereby oppress the function of the LDHA.

## Introduction

Lactate dehydrogenase (LDH, EC 1.1.127) is a family of 2-hydroxy acid oxidoreductases that catalyzes the reversible interconversion of pyruvate and lactate in the presence of the coenzyme NADH^1,2^. This reaction is the last step of glycolysis when limited amount of oxygen (O_2_) is available and is a principal way to regeneration NAD^+^ which is needed as a receptor to preserve cytosolic glucose catabolism^3^. Lactate dehydrogenase is a tetrameric enzyme composed of two major subunits LDHA and LDHB which can assemble into five different isoenzymes as H4, MH3, M2H, M3H, and M4. These isoenzymes (from the anode to cathode), according to their electrophoretic mobility, are referred to as LD1, LD2, LD3, LD4, and LD5, respectively^4^. LDHA (LDH5, M-LDH or M4) is predominantly found in anaerobic tissues like the skeletal muscle and the liver. LDHA needs a higher pyruvate concentration for the maximum enzyme activity. It means that the Michaelis constant (Km) of LDHA for pyruvate is 3–10 fold greater than the Km calculated for the LDHB form. LDHB (LDH1- H-LDH or H4) is predominantly found in aerobic tissues such as cardiac muscle^5^.

The human LDHA gene is located on short p arm of chromosome 11 (11p15.4)^6^. Its promoter region was determined as a direct target gene for the major transcription factors such as hypoxia-inducible factor I (HIF I) and c-MYC. These transcription factors are responsible for regulating the expression of several genes which are involved in vital biological processes such as cell proliferation, migration, angiogenesis, apoptosis, and glucose metabolism^7,8^ and they play a crucial role in adaptive responses of the cells to changes in the oxygen level^9^.

The low level of oxygen is a common feature of the most tumors called hypoxia which is related to the massive proliferation of cancer cells and also the expansion of the tumor tissue in the absence of an efficient vascular bed^10^. This phenomenon in which the metabolic pathway shifts from the oxidative phosphorylation (OXPHOS) toward the aerobic glycolysis is called Warburg effect reported in 1925 for the first time. In Warburg effect, LDHA is the most important factor playing a pivotal role in this metabolic shifting^11^.

Based on these findings, lactate dehydrogenase A plays a crucial role in normal aerobic glycolysis as the overexpression of LDHA has been reported in highly glycolytic human cancers. In hypoxia condition, observed in many types of cancer cells, LDHA is transcriptionally upregulated by the transcriptional factors responsible for the hypoxic adaptation such as HIF I and c-MYC^8^.

According to these observations, LDHA can be a critical factor in metabolic alterations which are required for the growth and the proliferation of certain tumors. Therefore, in recent years, it has been known that a targeted therapy in cancer has been proposed to inhibit the activity of LDHA via either natural or synthetic compounds to attenuate the tumor progression and invasiveness. Manerba *et al*. identified galloflavin (GF) as novel inhibitor for lactate dehydrogenase. According to their result, galloflavin arrest aerobic glycolysis through binding to the free enzyme and inhibited LDH activity^12^. Similar results have been also reported for Quinoline 3-sulfonamides, oxamate, N-hydroxyindole and Epigallocatechin Gallate^13–16^. The structures of some of these molecules mimic the substrate and cofactor of LDHA, so these compounds are capable of inhibiting the enzyme through a competitive inhibitory mechanism.

Over the recent decade, peptides have been used as a new class of drugs for the treatment of cancers, diabetes, and cardiovascular diseases^17,18^. Regarding the advent of cutting-edge technologies in proteomics, a large number of protein-protein interaction (PPI) have been identified which have critical roles in biological pathways inside the cells. Therefore, inhibition of PPI could be considered as an efficient approach for target therapy in some diseases such as cancers. Such PPIs include hotspot residues forming binding pockets covering a wide surface area^18^. Peptides as naturally occurring molecules are more suitable for covering these types of targets than the small molecules because of their larger molecular size. In addition, several advantages of peptides such as low toxicity, safety issues, ease of synthesis, high target specificity, tumor penetrability, feasibility of chemical modification, and biocompatibility make them suitable drug candidates^19,20^.

In this study, we employed *in silico* methods to design novel peptides for the inhibition of protein-protein interaction in order to disrupt subunit association of lactate dehydrogenase A during the tetramerization process. Several computational techniques such as docking and molecular dynamics simulation were used in this study. These techniques could clearly explain every molecular details from conformational changes during enzyme activity to molecular binding phenomena in an enzyme-ligand system at atomic level^21–26^. We have performed an intensive structural investigation for the understanding of dynamics and conformational motions occurred in LDHA. Then, inhibitory peptides have been designed based on its active conformation and interaction interface between LDHA subunits. The inhibitory effect of the designed peptides was verified by the dynamic light scattering technique.

## Result

### Subunit interaction analysis

**Figure 1:**
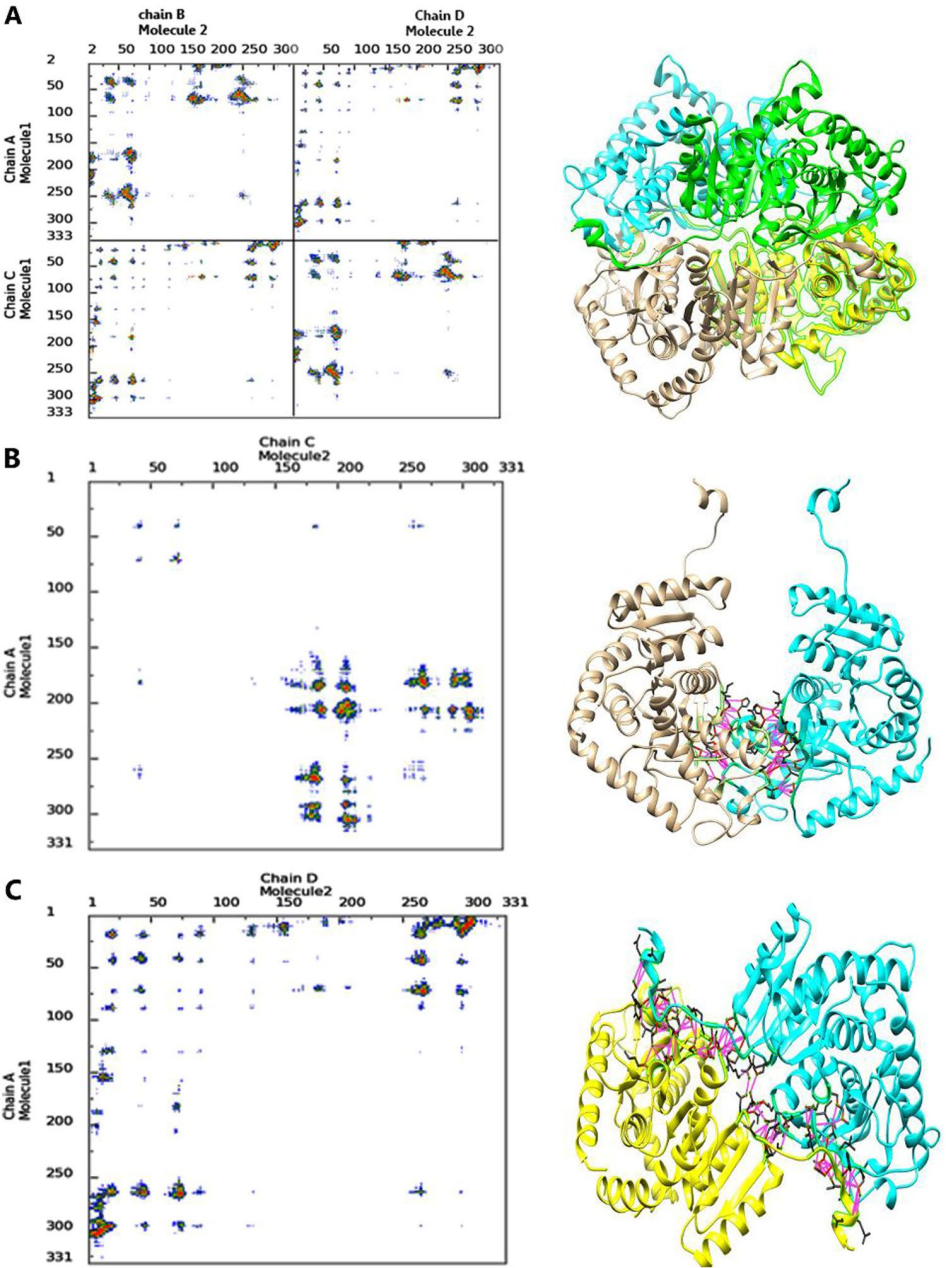
contact map and 3D structure of dimers and tetramer form of the enzyme. 3D structures of LDHA is depicted in the right as tetramer form (**A**), (**A**–**C**) dimer form (**B**), and (**A**–**D**) dimer form (**C**). In the left, the contact maps of the intermolecular contacts have been depicted as the colored dots. Red, yellow, green, and blue indicate contacts within 7 Å, 10 Å, 13 Å and 16 Å, respectively. Also violet displays hydrophilic-hydrophilic, green shows hydrophobic-hydrophobic and yellow shows hydrophilic-hydrophobic interactions.

**Figure 2:**
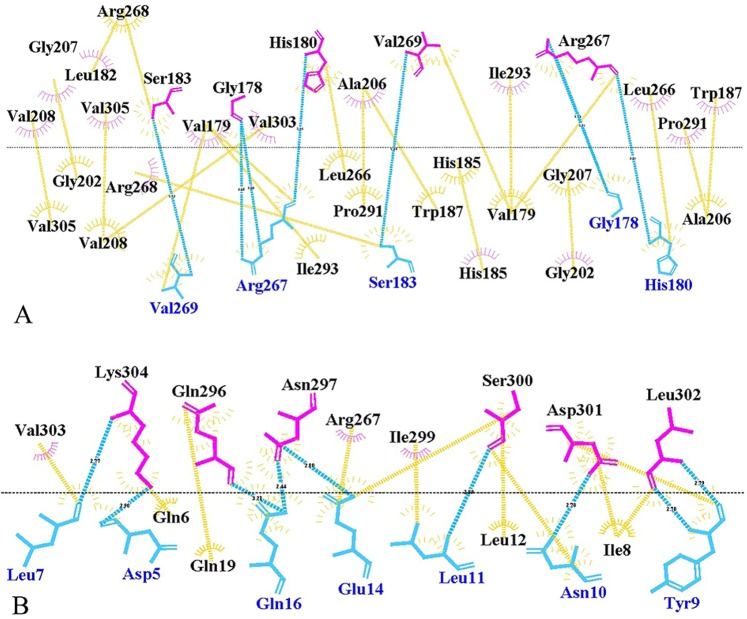
Two-dimensional scheme of interactions between different subunits of LDHA using LigPlot+. (**A**–**C**) Dimer form (**A**) and **A**–**D** dimer form (**B**). Hydrogen bonds and hydrophobic interactions are colored in blue and yellow lines respectively.

Lactate dehydrogenase A is a homotetrameric enzyme composed of four subunits as chains A, B, C, and D in the crystal structure. The assembly process of the enzyme is based on the interactions of these subunits to form an active complex of the enzyme. In this study, inhibitory peptides have been designed in order to disrupt the tetramerization process. Therefore, more detailed information about the intermolecular contacts during the formation of active enzyme was necessary. The assembly process of enzyme needs a dimer form as an intermediate structure which is built from A-C and B-D chains^27,28^. According to our results, the N-terminal residue of A and C chains in a dimer form of enzyme interacts with C-terminal residues of D and B chains, respectively to form a tetrameric structure. Figure 1 depicts tetrameric and dimeric forms of LDHA and its contact maps. COCOMAPS tool was used to study the distance between the residue pairs of a three-dimensional protein structure. The subunit contact map was derived using 8 Å cut-off value represented in the 2-dimensional binary matrix. In this study, LigPlot+ and Protein Interactions Calculator (PIC) server were used to demonstrate the hydrophobic interactions and hydrogen bonds formed between subunits (Fig. 2). Analysis of the interaction between two subunits A and C in dimer intermediate shows that, Val 179, Trp 187, Val 205, Ala 206, Val 208, Val 269, Pro 291, Ile 293, Val 303, and Val 305 of A chain contact with Val 269, Ile 293, Ala 206, Val 303, Trp 187, Pro 291, Val 305, Val 179, Val 205, Ala 206, and Val 208 from C chain by hydrophobic interactions within 5 Å. The A and C subunits have main chain-main chain hydrogen bonds between His 180 and Arg 267, and the main chain-side chains have hydrogen bonds in residues Gly 178 and Ser 183 with Arg 267 and Val 269. Based on these results, N-terminal residues of these subunits do not contribute to the formation of A-C and B-D dimers but it had an important role in enzyme tetramerization. The most important residues contributed in tetrameric interactions pertain to Leu 7, Ile 8, Tyr 9, Leu 11, Pro 74, Leu 266, Ile 293, Ile 299, Leu 302 and Val 303 of the A chain which contacts with Val 303, Ile 293, Leu 302, Ile 299, Leu 266, Pro 74, Ile 8, Leu 11, Tyr 9 and Leu 7 from the D chain by hydrophobic interactions within 5 Å. A and D subunits have main chain-main chains hydrogen bonds between Leu 7, Tyr 9, Leu 11, Leu 12, Ser 300 Leu 302, Lys 304 (A subunit) and Lys 304, Leu 302, Ser 300, Tyr 9, Leu 7 (D subunit). The main chain-side chain hydrogen bonds formed between residues Asp 5, Gln 16, Gln 296, Ser 300, Lys 304 from A subunit and Lys 304, Gln 296, Thr 17, Asn 10, Asp 5, Gln 6 from D subunit. All of the information showed that, N-terminal residues (5–17) and C-terminal residues (293. 296, 299 and 300–305) contact with each other during the enzyme assembly process.

### Molecular dynamics simulation of LDHA

**Figure 3:**
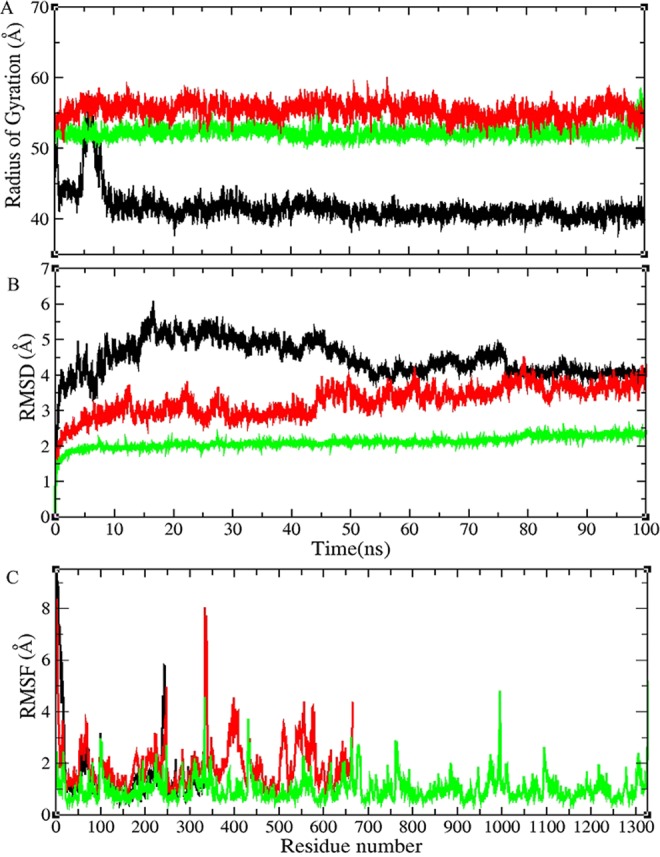
Molecular dynamics simulation of LDHA. The radius of gyration of LDHA (**A**), All-atom RMSD of LDHA (**B**), and RMSF of Cα atoms of LDHA (**C**). LDHA in the monomer (black line), (**A**–**D**) dimer (red line) and tetramer (green line) form of the enzyme were showed in three graph.

MD simulation of the human LDHA in the monomer, A-D dimer, truncated A-D dimer (deleted N-terminal arms) and tetrameric forms of the enzyme were carried out for 100 ns. A-C and B-D dimers have critical roles during assembly pathway of lactate dehydrogenase which dimerize to a tetrameric form via an N-terminal arms including residues 1 to 20^29^ (Schematic diagram are shown in Figure S1). In the tetrameric form, subunits A and D as well as B and C, interact with each other through the N-terminal arms which is important in activity and stability of the tetramer. Radius of gyration (R_gyr_) and Root mean square deviation (RMSD) of LDHA (monomer, A-D dimer and tetramer) were depicted in Fig. 3A,B.

The RMSD graph for monomeric and dimeric form of the enzyme showed higher value than the tetrameric forms during the simulation. The higher degree of fluctuations in the RMSD value of monomer form is related to local conformational rearrangement of enzyme during the simulation. According to the structural investigations during the RMSD raise, the most fluctuated regions are located at residues 1–7, 24–35, 165–185, 193–243, 295–310, and 320–332. The N-terminal (1–7) and C-terminal (320–332) regions are free and structural rearrangement in these regions do not contribute to the structural association. Residues 24–35, 165–185 and 193 which have important roles in LDHA’s activity as nucleotide binding site (29–57), substrate binding site (169) and proton acceptor residue are located in these regions respectively (Fig. S2).

The monomeric structure was more flexible during the simulation due to the large movement of the free N-terminal arm in the monomer, and the lower fluctuations were also observed in C-terminal region (residues 320–332), residues 55–72 and 225–248 which play critical roles in assembly process and LDHA’s activity respectively (Fig. S3).

The R_gyr_ graph for Cα atoms also showed a similar fluctuation for the monomeric form of the enzyme from 5 to 10 ns of simulation that is due to the structural rearrangement of the N-terminal arm of LDHA. Based on RMSD and R_gyr_ results, the tetrameric form of the enzyme shows a stable structure regarding monomer and dimer forms of LDHA. According to RMSF graphs for monomer, dimer (A-D), and tetramer, the amount of local flexibilities for monomer and dimer is higher than tetramer form especially in the residues 1–18, residues 55–72, residues 98–101 and residue 240–250 (Fig. 3C). Residues 7–18 and 55–72 are involved in the assembly process of the enzyme^30^. Other regions are associated with the NADH binding site (residues 99), substrate binding site (residues 106 and 248) and active site (residue 193). It seems that the difference in the flexibility pattern between the monomer, dimer and tetramer could justify the differences in the activity of these forms. These differences are significantly observed in N-terminal region 1–18 and also substrate binding site (248). This major fluctuation in residues 1–18 in the monomeric structure pertains to the free N-terminal arm which has no contact to its partner subunit which is responsible for the tetramerization process in tetrameric structure. In A-D dimer form, residues Val 179, His 180, Ser 183 Val 205, Ala 206, and Val 208 showed more flexibility than tetramer. These amino acids play critical roles in the interaction between two subunits A and C in dimer intermediate.

**Figure 4:**
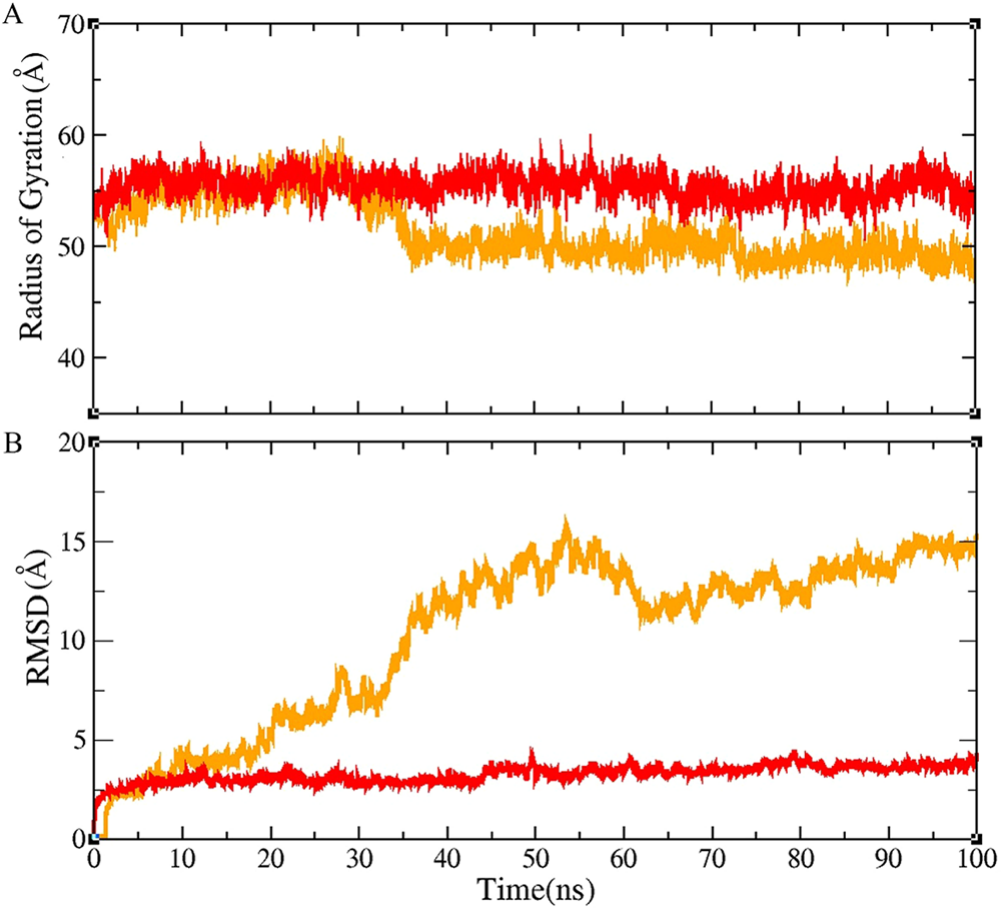
Molecular dynamics simulations of dimer (**A**–**D**) for wild type and truncated form. The radius of gyration of **A**–**D** dimer at two forms (**A**), All-atom RMSD of **A**–**D** dimer at two forms (**B**)  Dimer (**A**–**D**) in wild type and truncated form are shown in red and orange lines respectively.

In order to investigate the effect of the N-terminal arm in the stability of the association, residues 1–18 were removed from A-D dimer and MD simulation was carried out for 100 ns (Fig. 4). According to RMSD and R_gyr_ results, removing the N-terminal arms from A-D dimer resulted in a higher degree of fluctuations in this structure when compared to native dimer structure. NAPS was used for analysis of protein contact network to find more details about interaction between two subunits after removing the N-terminal arm. The results showed that the binding pattern was completely changed and structure reoriented in truncated dimer during the simulation which led to the creation of non-specific interaction between two subunits. In general, it can be concluded that the N-terminal arm acts as an anchor to maintain the position and distance of the two subunits relative to each other, and deletion of this region lead to considerable change in their orientation so that the subunits are connected in a different situation in the second half of the simulation (Fig. S4).

### Peptide design and molecular docking

According to MD results and interaction analysis of subunits, 18 residues in N-terminal do not involve in the dimeric (A-C) association, while blocking the interaction between N-terminal region of each monomer with the C-terminal region of its partner subunit (A-D and B-C dimers including residues 5–17 from N-terminal and residues 293, 296, 299 and 300–305 from C-terminal domain) can disrupt tetramerization of the enzyme and inhibit its activity. Based on this results, the N-terminal arm can be considered as an appropriate candidate to be used as template for preparation of peptide library. Therefore, a peptide library consisting of 46 peptides with 4, 5, 6, and 7 residues has been prepared (Table S1). Also, physicochemical properties of peptides were calculated by FAF drug and Toxtree servers. The selection of peptide sequences from the library was done based on the ability of the peptides to make appropriate contacts with the C-terminal residues of 300–305 as the main contact region for enzyme-peptide interaction. Then, GalaxyPepDock, PatchDock and FireDock servers along with AutoDock were applied to calculate the binding affinity of peptides to the tetramerization contact surface of LDHA residues of 300–305. According to docking result, peptides capable of mimicking the Tyr 9 and Asn 10 interaction from the N-terminal arm have been selected for the next step. This finding helped us to perform an evolutionary peptide design to those peptides containing Tyr and Asn as conserved residues.

**Figure 5:**
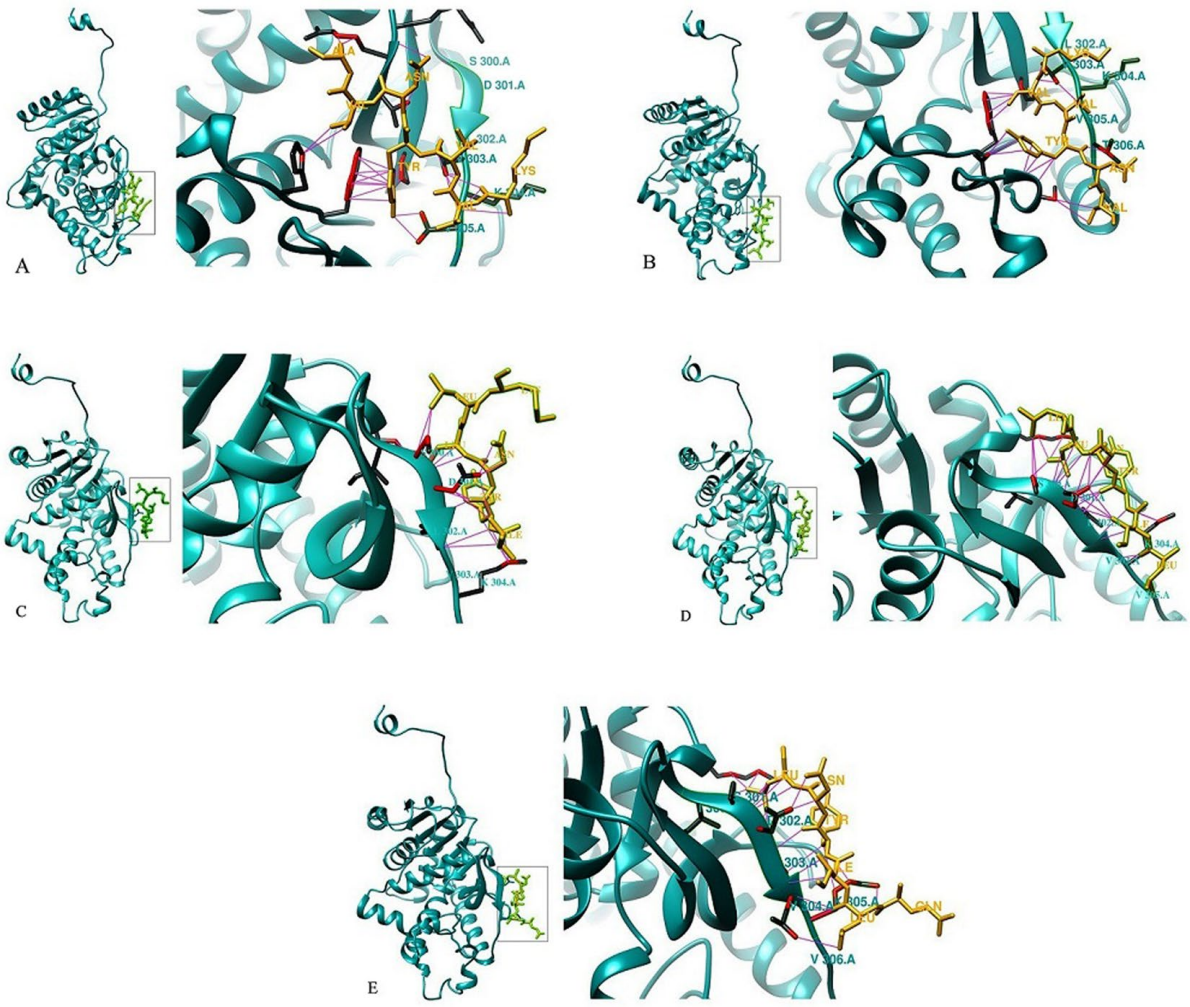
3D structure LDH-peptide complexes obtained from docking. Peptide KVVYNVA (**A**), peptide KVVYNV (**B**), peptide IYNLLK (**C**), peptide LIYNLL (**D**) and peptide QLIYNL (**E**).

Lastly, the selection of peptides was performed according to their binding energies and after that physicochemical properties of selected peptides were checked. Physicochemical properties of final peptides are reported in Table 1. Five final peptides are including KVVYNVA, KVVYNV, QLIYNL, LIYNLL, and IYNLLK with binding energies of −27.32 kcal.mol^−1^, −26.35 kcal.mol^−1^, −29.47 kcal.mol^−1^, −33.25 kcal.mol^−1^, and −28.67 kcal.mol^−1^, respectively. The binding conformations of selected peptides depicted in Fig. 5. According to the results, Leu 2, Ile 3, Tyr 4 and Leu 6 from peptide QLIYNL have hydrophobic interaction with Leu 279, Ile 293, Ile 299, Leu 302 and Val 303 from enzyme subunit within 5 Å cut-off. The side chains hydrogen bonds formed between residues Gln1, Leu 2, Tyr 4, Leu6 and Ser 300, Leu 302, Lys 304.

**Table 1:** Physicochemical properties of final peptides.

	Number of residues	MW g/mol	Net charge at pH 7	Iso-electric point:	LogP	LogSw	logD
KVVYNVA	7	791.93	1	9.55	−2.15	−1.91	−7.41
KVVYNV	6	720.86	1	9.55	−4.07	−0.39	−6.93
QLIYNL	6	762.89	0	3.32	−1.75	−2.05	−4.00
LIYNLL	6	747.92	0	3.57	−0.36	−2.83	−1.40
IYNLLK	6	762.94	1	9.75	−0.82	−2.50	−2.61

Leu 1, Ile 2, Tyr 3 and Leu 5 from peptide LIYNLL have hydrophobic interaction with Ile 293, Ile 299, Leu 302, Val 303, within 5 Å cut-off. The side chain and main chain hydrogen bonds are formed between Leu 1, Tyr 3, Asn 4, Leu 5 and Ser 300, Asp 301, Leu 302, Lys 304. Peptide IYNLLK has similar interaction pattern. Removing the Leu 1 from peptide LIYNLK led to a decrease in the interaction between peptide IYNLlK and Val 303 and Lys 304 when compared to LIYNLL. Docking results for three peptides showed that peptide LIYNLL had the best binding energy and the binding energies for two other peptides (QLIYNL and IYNLK) were almost the same.

Val 2, Val 3, Tyr 4 and Val6 from Peptide KVVYNVA had hydrophobic interaction with Trp 187, Val 269, Pro 291, Val 303 and Val 305 from enzyme within 5 Å cut-off, also Side chain and main chain hydrogen bond was observed between Lys 1, Asn 5 and Arg 297, Lys 304. Val 2 and Val 3 from peptide KVVYNV had hydrophobic interactions with Trp 187, Val 269, Pro 291, and Val 303 within 5 Å cut-off and also a hydrogen bond was observed between Tyr 4 and Thr 306. According to the docking results, peptide KVVYNVA has lower binding energy than peptide KVVYNV. According to the log p, peptides KVVYNVA and KVVYNV were more hydrophilic (−2.15 and −4.07) when compared to other peptides.

### Analysis of binding free energies for LDH-peptide complexes

MM-PBSA method was used for the calculation of the binding energies for five peptides when bound to LDHA (Table 2). The best binding energies were obtained for LIYNLL and QLIYNL with −17.6370 ± 0.5236 kcal.mol^−1^ and −13.2642 ± 0.4368 kcal.mol^−1^, respectively. According to MM-PBSA analysis, electrostatic interactions in binding affinities in LIYNLL and QLIYNL has a greater role compared to the other three peptides however, van der Waals interaction energy also plays a critical role in peptide binding to the LDHA. Peptides KVVYNV and IYNLLK had the same binding free energies (−12 kcal.mol^−1^) and the lowest binding affinity obtained for KVVYNVA with −6.5899 ± 0.4620 kcal.mol^−1^.

**Table 2:** The MM-PBSA binding energies (kcal·mol^−1^) for the five complexes of LDH-peptide.

	Δ*G*_bind_	ENPOLAR	EPB	EEL	VDWAALS
KVVYNVA	−6.5899 ± 0.4620	−24.7332	2.9453	1.1994	−34.3666
KVVYNV	−12.2643 ± 0.5234	−30.2444	8.0891	−4.9134	−37.7497
QLIYNL	−13.2642 ± 0.4368	−30.1603	26.5104	−25.0633	−38.0179
LIYNLL	−17.6370 ± 0.5236	−34.2421	19.7448	−17.6057	−44.9546
IYNLLK	−12.0756 ± 0.3503	−29.6473	7.2210	−3.3950	−38.6558

The binding energies of hexa-alanine peptide was calculates using MM-PBSA method as negative control. The results showed that the peptide binding to the enzyme was weak in the way that it was detaches from enzyme during the simulation.

### Peptide synthesis and dynamic light scattering analysis

**Figure 6:**
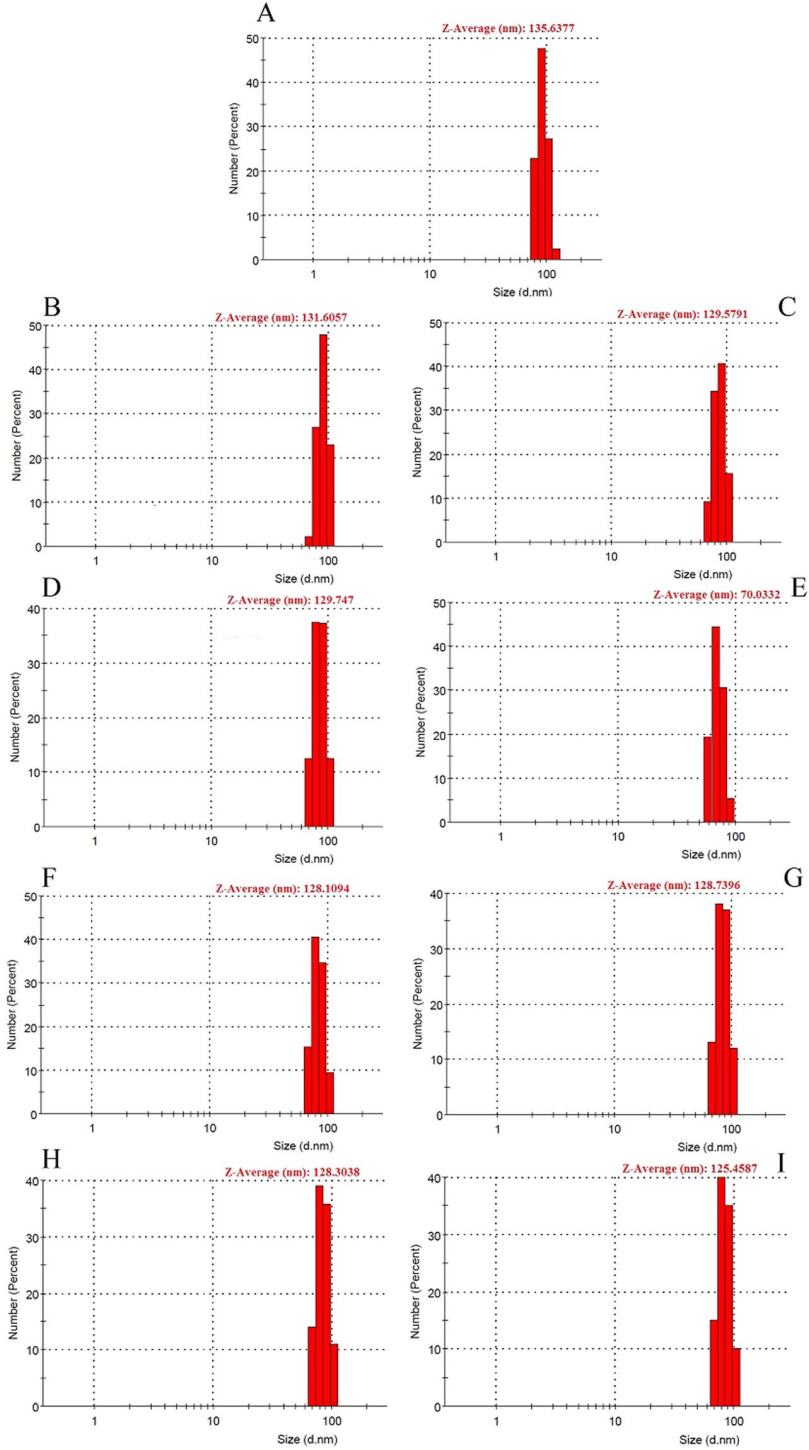
Size distribution of lactate dehydrogenase measured by DLS experiment. The graphs show the changes in size distribution of lactate dehydrogenase in the absence and the presence of peptides. Enzyme in the absence of peptide in substrate solution (**A**), enzyme in substrate solution in the presence of KVVYNVA at 5.5 and 8 μM (**B**,**C**), enzyme in substrate solution in the presence of KVVYNV at 5.5 and 8 μM (**D**,**E**), enzyme in substrate solution in the presence of QLIYNL at 5.5 and 8 μM (**F**,**G**), enzyme in substrate solution in the presence of IYNLLK at 5.5 and 8 μM (**H**,**I**).

Based on the *in silico* studies, five peptides were selected for the DLS analysis. After peptide synthesis, dynamic light scattering technique was employed in order to observe the effect of the designed peptides to disrupt the protein-protein interaction in the tetrameric form of the enzyme. The size distribution of LDHA in the substrate solution either in the presence or the absence of peptides was reported in Fig. 6. Regarding the DLS results, the average size distribution for LDHA in the absence of peptides was 135.63 nm. Peptide KVVYNVA with lowest binding affinity (−6.5899 ± 0.4620 kcal.mol^−1^) did not change the LDHA average size distribution in 5.5 and 8 µM concentrations. Peptides KVVYNV, QLIYNL and IYNLLK with similar binding free energies (−12.2643 ± 0.5234 kcal.mol^−1^, −13.2642 ± 0.4368 kcal.mol^−1^, −12.0756 ± 0.3503 kcal.mol^−1^) showed different behavior during the assessment of average size distribution. The average size for LDHA in the presence of these peptides at the concentration of 5.5 µM was slightly decreased. Increasing the peptide concentration to 8 µM revealed a significant decrease in the average size of LDHA in the presence of KVVYNV when compared to enzyme solution without peptide as a control. Therefore, peptide KVVYNV was successful to disrupt LDHA tetramerization. In contrast, average size for LDHA was not significantly decreased in the presence of two other peptides (IYNLLK, QLIYNL). Since aggregation was observed for these two peptides, this might effect on their ability to bind to the enzyme. Despite the best binding free energy for LIYNLL peptide, limited solubility of theses peptide in water solution could effect on their ability to bind to the enzyme population adequately. Since the expected result was not obtained for the peptide IYNLLK, the molecular weight measurement for the LDHA has been done again in the presence and absence of the peptide IYNLLK.

The LDHA molecular weight is 35 kDa, 70 kDa and 140 kDa for monomer, dimer and tetramer forms, respectively. Calculating the molecular weight of enzyme in a substrate solution in the presence and absence of peptides was reported in Figure S5. According to the results, the average molecular weight of enzyme in the absence of peptide IYNLLK was 244 ± 81 kDa (R^2^ = 0.977). The average molecular weight for the enzyme in the presence of peptide at the concentration of 8 µM was 97.7 ± 56.6 kDa (R^2^ = 0.591). Therefore, the average molecular weight for the enzyme in complex with the peptide was decreased when compared to the enzyme in the absence of peptide.

## Discussion

The metabolic profile of tumor cells is significantly different from the normal cells although they are present in the same tissue or organ^31^. Such discrepancies cast doubt that cancer should be classified as the metabolic diseases. The altered metabolism program of tumor cells is attributed to a higher growth rate of cells which needs an excess amount of bioenergetics and biosynthetic demands to perpetuating cell proliferation^31^. With regard to the difference in metabolic profile, researchers are able to discover novel molecules for target therapy in cancer.

In 1925, Warburg discovered a remarkable distinction between the relative uses of the various modes of energy produced in normal and tumor cells. In normal tissues, tricarboxylic acid (TCA) cycle and oxidative phosphorylation are the routine modes for pyruvate catabolism obtained from the glycolysis pathway. In contrast to normal cells, the rate of aerobic glycolysis is dramatically increased in cancer cells and the majority of pyruvate is converted to lactic acid during the energy production^11^. The critical enzyme in aerobic glycolysis is lactate dehydrogenase (LDH) that can be an appropriate candidate for target therapy according to Warburg effect. Lactate dehydrogenase is a tetrameric enzyme consisted of two types of subunits namely LDHA and LDHB. The maximum activity of LDH needs a quaternary structure obtained from the interactions between subunits. This enzyme is a dimer of two dimers and the assembly process of the tetrameric enzyme is created by the intermediate assembly of a dimer formation^27,28^.

Regarding the importance of lactate dehydrogenase in the metabolism of the cancer cells^32^, several inhibitors have been proposed to oppress the activity of LDHA. These inhibitors are composed of natural and synthetic molecules which are similar to the substrate or cofactor structures and mimic their interaction with the enzyme. These inhibitors such as 3-((3-carbamoyl-7-(3, 5-dimethylisoxazole-4-yl)−6-methoxyquinolin-4-yl) amino) benzoic acid were characterized as an NADH-competitive LDHA inhibitor^13^. Galloflavin is another inhibitor that suppresses the human LDH isoforms by favorably binding the free enzyme, without competing with the substrate or cofactor^12^. N-hydroxyindole-based is another inhibitor of LDHA which contests with both the substrate (pyruvate) and cofactor (NADH) interaction^15^. Since no molecule has been designed to hinder the assembly of the tetramerization of LDH so far, no peptide has been accordingly designed to show such effect. In this study, we used computational methods to design novel peptide inhibitors which are capable of disrupting the assembly process of tetramerization.

Therefore, we employed molecular dynamics simulation in order to obtain more information about the structural dynamics and interactions of enzyme subunits in different structural combinations (monomer, dimer, and tetramer). Our result suggested that subunits A-C and also B-D interact with each other via C-terminal residues to form an intermediate product but the N-terminal residues are not involved in this interaction. The critical interactions leading to the formation of the tetramer, are located in the N-terminal arm (residues 5–17) of each subunit contacting the residues 293. 297, 299 and 300–305 in C-terminal of other subunits (A-D and B-C and vice versa).

According to the simulation data, the N-terminal arm in monomer has a highly fluctuated structure while the flexibility of this region is dramatically reduced in tetramer form. Since N-terminal regions of each monomer have a critical role in the formation of the tetramer, residues 5–17 keep their interactions with partner subunits (293. 296, 299 and 300–305) to stabilize the tetramer form.

Several studies have been conducted which are in agreement with our results about the role of the N-terminal region in the enzyme tetramerization. Read *et al*. in 2001 reported that the small changes in N-terminal region play a pivotal role in the association of subunits between the tetramers of H and M forms of human lactate dehydrogenase^33^. Zheng *et al*. in 2004 investigated the importance of N-terminal region of subunits in rabbit muscle lactate dehydrogenase stability^34^. Their result showed that N-terminal deletion caused a reduction in the enzyme activity and made it more sensitive to denaturing environment. A Similar report has been also published by Schmidt *et al*. in 1999. They reported that the subunit A and C interact with each other to create an intermediate product but 18 residues of N-terminal do not participate in any interaction during this process^30^. According to above statements, we suggested that the effect of peptide inhibitory is mediated through an interaction of A-D and B-C dimers in tetramer region of the C-terminal domain.

At first, we provided a library of peptides and then, binding free energies of these peptides were calculated. Based on docking results, there were two key residues (Tyr 9 and Asn 10) in N-terminal region playing a critical role in the interaction. Seemingly, the highest binding affinity was related to peptides possessing Tyr and Asn as conserved amino acids. The similar result was reported by Fan *et al*. in 2011^35^. With respect to their report, phosphorylation at Y10 and Y83 improves the LDHA activity thereby facilitating the formation of active, the tetrameric form of LDHA, and the binding of substrate (NADH) to LDH, respectively. Moreover, Y10 phosphorylation of LDHA is common in diverse human cancer cells attributed to the activation of multiple oncogenic tyrosine kinases.

In the second step, we selected five peptides according to the docking results. Also, the appropriate binding position of peptides to the enzyme was considered. Peptides QLIYNL, LIYNLL, and IYNLLK interact in a suitable orientation in which the key residues at the C-terminal were covered successfully when compared to two other peptides (KVVYNVA and KVVYNV). We also calculated the binding free energy of these five peptides via MM-PBSA method. Peptide LIYNLL has the highest binding affinity along with appropriate binding position from both docking and MM-PBSA calculations.

Finally, in order to examine the inhibitory effect of peptides, the average size distribution and molecular weight were measured by DLS to estimate the tetramer and dimer populations of LDHA in the presence of peptides. The average size distribution of LDHA was decreased in the presence of KVVYNV and the average molecular weight (Mw) of LDHA was also decreased upon the addition of IYNLLK. Notably, according to the average molecular weight of enzyme, the percentage of dimer population was also decreased in the presence of IYNLLK suggesting that this peptide can affect the enzyme tetramerization process.

## Conclusion

Lactate dehydrogenase A plays an important role in metabolic pathways of the cancer cells. The overexpression of enzyme has been reported in many malignant tumors and associated with the growth and proliferation of the tumors. Therefore, the inhibition of LDHA activity may provide an opportunity for anti-cancer agents to interfere with tumor growth and invasiveness. Other study suggested that the inhibition of LDHA was also found to be a way to overcome the acquired resistance of breast cancer cells to Taxol^36^ and trastuzumab^37^. Therefore, the oppression of LDHA can be a potential target for the treatment of cancer.

Our results provide a model for LDHA inhibition by the novel designed peptides in which they destabilize the interacting subunits. Several advantages of peptide like low toxicity, ease of synthesis, and high target specificity make them as a novel anti-cancer agent for cancer therapy process. In this study, the inhibitory peptides mimic the tetramerization site on the N-terminal region from one subunit and C-terminal domain from partner subunit. These inhibitors had a great impact on enzyme assembly process thereby inactivation of the enzyme. *In vivo* investigation of these peptides on cancer cell lines will be done in our future experiments.

## Methods

### Structural investigation of LDHA

Crystal structures of the human muscle L-lactate dehydrogenase in apo, ternary and inhibitor-bound forms (PDB code: 4ojn) and ternary complex with NADH and oxalate (PDB code: 4okn) were obtained from Protein Data Bank (https://www.rcsb.org). Structural investigations were performed by Swiss-PDB viewer 4.0.1 and Pymol 1.3^38,39^.

### Intermolecular interactions of LDHA subunits

Structural analyses of LDHA were performed by Swiss-PDB viewer and PDBsum in order to explore the key role of residues involved in tetramerization of four subunits. COCOMAPS was used for the analysis of intermolecular contact maps^40^. In the contact map, each contact was colored according to the physicochemical nature of the two interacting residues. Protein Interactions Calculator (PIC) server (http://pic.mbu.iisc.ernet.in) and LigPlot+ were used for protein-ligand interaction analysis. This program is applicable for depicting schematic diagrams of hydrogen and hydrophobic contact in protein-ligand complex^41,42^.

### Molecular dynamics (MD) simulations

AMBER14 package^43^ along with ff14SB force field^44^ was used for molecular dynamics (MD) simulation of LDHA in four forms of monomer, A-D dimer, truncated A-D dimer (deleted N-terminal arms) and tetramer. Neutralization of complex was done by the addition of Cl^−^ ions to the structure. Solvation of structures were performed by *xLEaP*^43^ (Amber Tools 15) with 10 Å layer of explicit water TIP3P model in a truncated octahedral box. The coordination and the topology files were then saved for the next steps of minimization and MD simulations. Energy minimization of the solvated LDHA was done in two steps. At first, water and ions were minimized with 3000 steps. The whole system was energy-minimized for 5,000 steps each using steepest-decent and conjugate gradient algorithms. The cutoff distance was set to 10 Å for the calculation of non-bonded interaction by PME method in the periodic boundary condition.

The heating of system was done from 0 to 300 K for a period of 200 ps, with the NVT ensemble using Langevin thermostat with collision frequency of 2 ps^−1^ ^45^. The SHAKE algorithm used to constrain bonds involving hydrogen atoms^46^. Before production MD, the equilibration was done for 1 ns in the NPT ensemble. The pressure was set to 1 atm using Berendsen barostat with relaxation time 2 ps. Eventually, MD simulation were carried out for 100 ns with the NPT ensemble. The time step was adjusted to 2 fs and the coordinates were saved every 0.8 ps^21^.

### Trajectory analysis

Analysis of the trajectories has been done by *cpptraj*^47^ from Amber tools 15 for calculating the root mean square deviation, fluctuation, and radius of gyration.

### Network analysis of protein structures

NAPS server (http://bioinf.iiit.ac.in/NAPS/) was used to investigate the network Analysis of Protein Structures at different snapshots from the simulation^48^.

### Peptide design

Peptides have been designed based on peptidomimetics and *in silico* design. First of all, we performed MD simulations to observe conformational changes in a monomer, dimer, and tetramer. Then, interaction patterns have been investigated to fully understand protein-protein interaction during the MD simulation to extract the most populated conformation. Then, we used the most stable structure for the peptide design. Peptidrive^49,50^ from Rosetta server^51^ was used for designing peptides based on the protein-protein interaction pattern. Then a peptide library has been prepared containing the N-terminal arm (7–12) and other sequences obtained from peptidrive. Finally, several peptide derivatives from these sequences generated by replacing residues based on the similarity of their side chains.

### Docking of peptide

Local docking of peptides with monomer form of LDHA was done by AutoDock 4.2^52^. AutoDockTools 1.5.6 was used for preparation of LDHA and peptides. Polar hydrogens were added and Gasteiger charges were calculated. The torsion tree was specified and molecules were saved in the PDBQT format and used for the docking. The grid map was fixed to 52 × 52 × 50 Å along the x, y, and z-axes, respectively on residues 300–305 from the C-terminal domain of LDHA. The Lamarckian genetic algorithm (LGA) was employed using the default parameters. Docking was conducted on a rigid receptor and peptides were considered flexible. The docking tests repeated at least three times to obtain converged results. Then, the binding modes of the complexes were investigated by AutoDockTools, Swiss-PDB viewer, LigPlot and, Pymol. GalaxyPepDock, PatchDock and FireDock also were used for protein-peptide docking. The GalaxyPepDock server constructs models via energy-based optimization that approves for structural flexibility^53^. The method of FireDock^54^ server is based on fast rigid-body docking algorithms.

### Investigation of the physicochemical properties of peptides

In order to find the best interacting peptides, a peptide library was designed and then the physicochemical properties of the peptides were analyzed via toxtree servers for the selection of the best peptides^55^. Then, a set of the most suitable peptides were chosen based on the docking results along with physicochemical properties. The Molecular weight, isoelectric point,  logP,  logSw, and  logD of the selected peptides were measured by FAF-Drugs3^56^.

### Molecular mechanics Poisson–Boltzmann surface area (MM-PBSA) calculation

After peptide selection, LHDA-peptide complexes were used for MD simulations by Amber 14 for 50 ns using ff99SB force field. The calculation of binding free energies was done by *mmpbsa*.*py*^57^. We used 200 frames of each trajectory for the calculation of the final Δ*G*_bind_ values.

### Peptide **synthesis and dynamic light scattering** assay

The best peptides were synthesized by BIO BASIC Company (purity > 95%). Then, peptide solutions were prepared according to amino acids characteristics. Three peptides with the following sequence KVVYNVA, KVVYNV, IYNLLK were dissolved in aqueous buffer PBS (Phosphate-buffered saline) and two peptides (QLIYNL and LIYNLL) consisting of hydrophobic amino acids were dissolved in the least possible volume of DMSO in water as 50% (v/v).

Dynamic light scattering (DLS) has been done by ZEN5600 Zetasizer (Malvern Instruments Ltd) for the size distribution and molecular weight detection of lactate dehydrogenase. DLS data were obtained at room temperature (25 °C) with the concentrations of 6 × 10^−4^ M for pyruvate and 18 × 10^−4^ M for NADH as a substrate solution. The concentration of enzyme was adjusted to 0.5 mg/ml in the presence of peptides at two concentrations (100 and 150 µM) and then enzyme-peptide solution reached to the final volume of 1 mL by substrate solution So final concentration of peptide were 5.5 and 8 µM.

Size distribution graphs were used for depicting the relative amounts of monomer, dimer, and tetramer forms of the enzyme in the sample. The average value of molecular weight was reported as Debye plot which was performed as an intensity of scattering light (KC/RoP (1/kDa)) against the centration (mg/mL). This test was carried out in the same conditions as size distribution assay and the enzyme-peptide solution was prepared according to previous step. The final concentration of peptide in enzyme-peptide solution was 8 µM that was selected according to size distribution results.

The intercept of the plot provides 1/MW and the slope is applied for calculating the second virial coefficient. The dñ/dC (differential refractive index increment) values of 0.183 and 0.186 mL/g were used for the MW assay of the enzyme and enzyme with peptide, respectively.

## Supplementary information

supplementary
