# Supplementary material for: Novel Peptide Inhibitors for Lactate Dehydrogenase A (LDHA): A Survey to Inhibit LDHA Activity via Disruption of Protein-Protein Interaction
Source: Sci Rep. 2019 Mar 18;9:4686. doi: 10.1038/s41598-019-38854-7 (PMC6423238; doi:10.1038/s41598-019-38854-7)
Supplement: Supplementary file 1 — supplementary [file 41598_2019_38854_MOESM1_ESM.docx]

**Novel Peptide Inhibitors for Lactate Dehydrogenase A (LDHA): A Survey to Inhibit LDHA Activity via Disruption of Protein-Protein Interaction**

Farzaneh Jafary^1^, Mohamad Reza Ganjalikhany^2^*, Ali Moradi**^1ǂ^**, Mahdie Hemati^1^, Sepideh Jafari^2^

1. Department of Clinical Biochemistry, Faculty of Medicine, Shahid Sadoughi University of Medical Sciences, Yazd, Iran.

2 Department of Biology, Faculty of Sciences, University of Isfahan, Isfahan, Iran

* Corresponding author: Mohamad Reza Ganjalikhany

Email: [m.ganjalikhany@sci.ui.ac.ir](mailto:m.ganjalikhany@sci.ui.ac.ir)

Tel: +98-31-37932250, P.O. Box: 81744

ǂ Corresponding author: Ali Moradi

Email: [morady2008@gmail.com](mailto:morady2008@gmail.com)

Tel: +98-351-8202633

**Supplementary Figures**

**
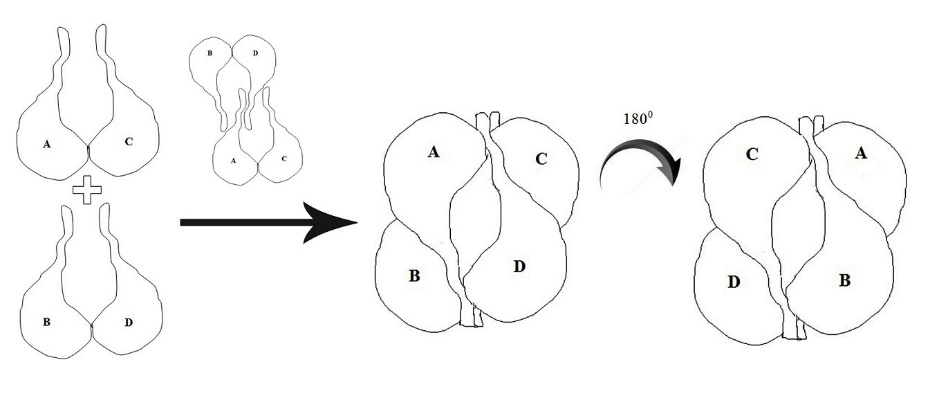
**

**Figure S1**. **Schematic diagram of the late stages of the major assembly pathway of LDHA**. Assembly starts by interaction between two subunits A-C and B-D. Then dimerization happens between two intermediate dimer structures by the N-terminal arms and creates a stable tetramer structure.


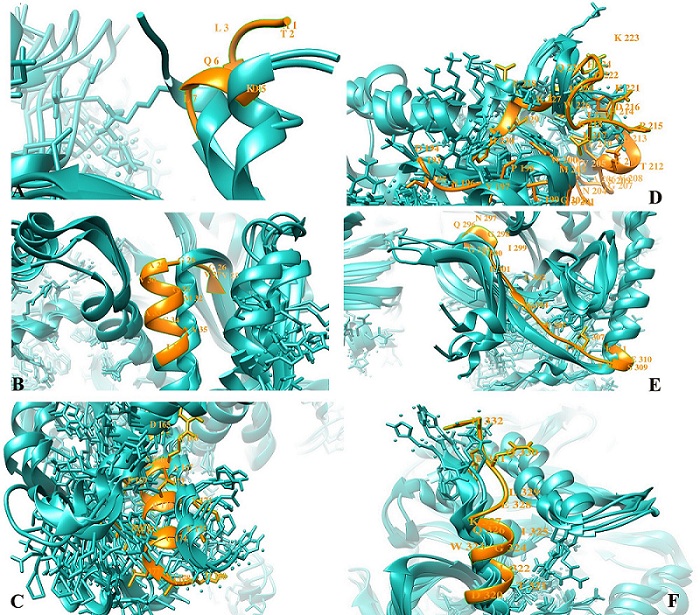


**Figure S2.** **Comparison between LDHA structures at different times (42, 44, 46 and 48 ns) during simulation.** The amino acids with the highest movements are residues 1-5 (A), residues 24-35 (B), residues 165-185 (C), residues 193-243 (D), residues 295-310 (E) and residues 320-332 (F).

**
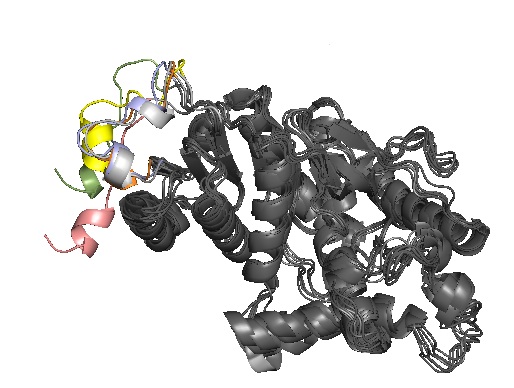
**

**Figure S3.** **Comparison between monomer forms LDHA at different times** **during the simulation.** Movements of the free N-terminal arm are shown by different colors.


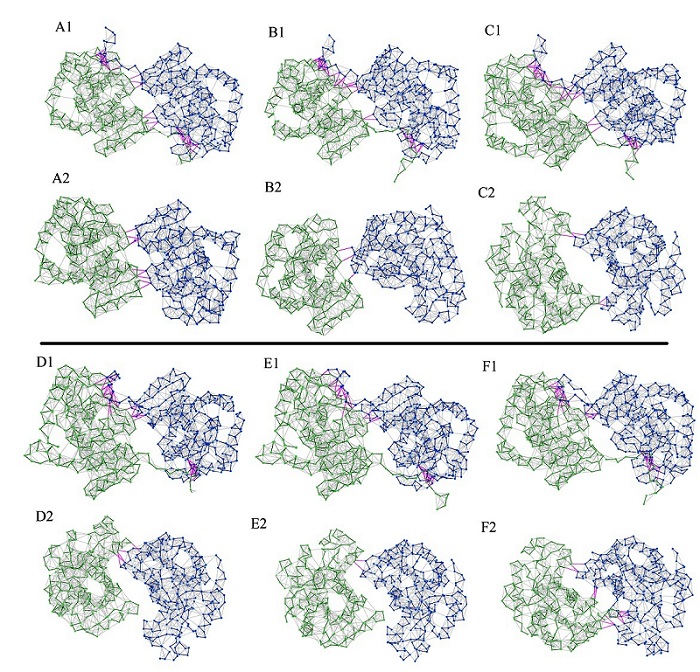


**Figure S4. Contact network analysis of native and truncated dimer (A-D) during the simulation.** The structure of native dimer is shown in figures A1, B1, C1, D1, E1 and F1 and truncated dimer is shown in figures A2, B2, C2, D2, E2 and F2. As depicted in the initial frame of simulation for native dimer (figure A1), in addition to the N-terminal arms, other residues such as Met 40, Asp 45, Thr 73, Pro 74, Lys 264, Asn 265 and Leu 266 also participated in the interaction between two subunits. These residues are poorly involved in the interaction at the native structure and this pattern was slightly changed during the simulation. In truncated A-D dimer, these residues lost their connections and two subunits made non-specific interaction during the simulation (Fig B2, D2 and E2). In this case, residues Pro 18, Gln 19, Lys 42, Ala 168, Arg 169, Tyr 172, Pro 182, and Leu 183 are involved in which do not play any role in formation of native A-D dimer.


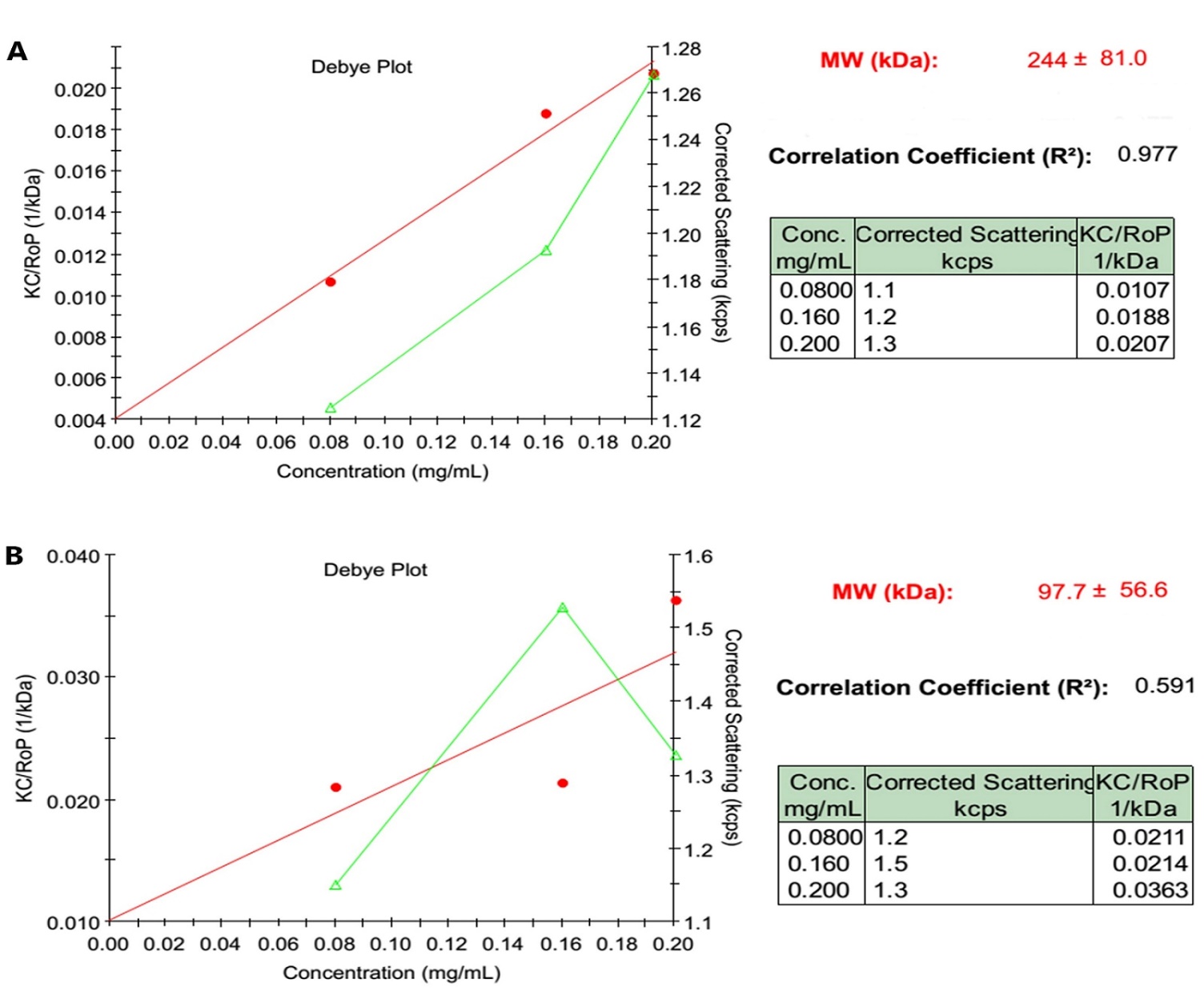


**Figure S5**. Debye plot of variation in the average intensity of scattering light (KC/RoP (1/kDa)) against concentration (mg/mL), LDH enzyme in the absence of peptides (A), LDHA in presence of IYNLLK peptide.

**Supplementary Table**

**Table S1. The library of designed peptides**

| Firedock | Auto  Dock | Average hydrophilicity | Iso-electric point | Net charge at pH 7 | MW  g/mol | Number of residues | Sequence |
| --- | --- | --- | --- | --- | --- | --- | --- |
| -1.94 | 4.2- | -1.4 | 5.9 | 0 | 521.61 | 4 | IYNL |
| -22.64 | -4.72 | -1.4 | 5.9 | 0 | 521.61 | 4 | YNLL |
| -19.3 | 4.84- | -1.4 | 5.9 | 0 | 521.61 | 4 | LIYN |
| -18.6 | -4.25 | -0.1 | 3.1 | -1 | 473.53 | 4 | DNLL |
| -19.86 | -4.34 | -0.1 | 3.3 | -1 | 487.55 | 4 | ENLL |
| -18.72 | -3.51 | 1.5 | 6.9 | 0 | 500.55 | 4 | EQKP |
| -16.47 | -4.87 | 0.9 | 3.3 | -1 | 445.43 | 4 | ESNP |
| -20.3 | -2.73 | -0.1 | 10.1 | 1 | 486.61 | 4 | KNLL |
| -18.13 | -3.51 | -0.1 | 11.1 | 1 | 514.63 | 4 | RNLL |
| -17.97 | -4.15 | -0.2 | 9.7 | 1 | 536.63 | 4 | YNKL |
| -16.2 | -1.82 | -0.9 | 5.9 | 0 | 495.53 | 4 | YNLS |
| -14.47 | -2.31 | -0.8 | 5.9 | 0 | 513.57 | 4 | YNMS |
| -18.59 | -7.65 | -1 | 5.9 | 0 | 505.57 | 4 | YNPL |
| -18.07 | -6.26 | -0.4 | 5.9 | 0 | 479.49 | 4 | YNPS |
| -13.97 | -4.06 | -0.9 | 5.9 | 0 | 495.53 | 4 | YNSL |
| -17.86 | -2.1 | -0.7 | 9.9 | 1 | 563.70 | 4 | YRLL |
| -28.18 | -2.76 | -1.5 | 5.9 | 0 | 634.77 | 5 | LIYNL |
| -20 | -2.57 | -1.5 | 5.9 | 0 | 634.77 | 5 | IYNLL |
| -22 | -3.4 | -0.4 | 9.9 | 1 | 649.75 | 5 | VVYNR |
| -24 | 4.5- | -1.3 | 5.9 | 0 | 592.69 | 5 | VVYNV |
| -19.5 | -4.02 | -0.8 | 5.9 | 0 | 522.56 | 5 | AVYNG |
| -21.63 | -3.54 | -1 | 5.9 | 0 | 550.61 | 5 | VGYNV |
| -10.8 | -1.88 | -1 | 5.9 | 0 | 626.73 | 5 | MYNLS |
| -20.51 | -4.44 | -1.2 | 5.9 | 0 | 642.71 | 5 | YNLSF |
| -18.54 | -2.35 | -1 | 5.9 | 0 | 626.73 | 5 | YNLSM |
| -18.51 | -3.42 | -0.7 | 5.9 | 0 | 582.61 | 5 | YNLSS |
| -17.24 | -4.87 | -1.1 | 5.3 | 0 | 642.73 | 5 | YNPCF |
| -17.2 | -4.57 | -0.7 | 5.9 | 0 | 592.65 | 5 | YNPLS |
| -17.91 | -5.1 | -0.9 | 5.9 | 0 | 626.67 | 5 | YNPSF |
| -16.97 | -3.87 | -0.5 | 7.8 | 0.1 | 616.63 | 5 | YNPSH |
| -16.26 | -4.52 | -0.3 | 5.9 | 0 | 607.62 | 5 | YNPSQ |
| -29.47 | -1.5 | -1.2 | 5.9 | 0 | 762.90 | 6 | QLIYNL |
| -33.25 | -2.25 | -1.6 | 5.9 | 0 | 747.93 | 6 | LIYNLL |
| -26.35 | -3.19 | -0.6 | 9.7 | 1 | 720.87 | 6 | KVVYNV |
| -25 | -2.08 | -0.6 | 9.7 | 1 | 734.89 | 6 | KLVYNV |
| -20.86 | -1.77 | -0.7 | 5.9 | 0 | 740.77 | 6 | YNPNSF |
| -15.71 | -1.83 | -1.0 | 5.9 | 0 | 739.83 | 6 | YNPLSF |
| -17.91 | -3.27 | -0.7 | 5.9 | 0 | 713.74 | 6 | YNPSFS |
| -17.04 | -4.99 | -0.6 | 5.9 | 0 | 706.75 | 6 | YNPSNL |
| -14.45 | -0.54 | -0.5 | 6 | 0 | 444.48 | 6 | AAAAAA |
| -34.36 | -0.48 | -1.3 | 5.9 | 0 | 876.06 | 7 | QLIYNLL |
| -28.67 | -0.96 | -0.8 | 9.7 | 1 | 762.95 | 7 | IYNLLK |
| -29.26 | - | -0.2 | 6.9 | 0 | 892.06 | 7 | IYNLLKE |
| -26 | 0.22 | -0.5 | 9.7 | 1 | 849.00 | 7 | QVVYNVK |
| -27.32 | -0.49 | -0.6 | 9.7 | 1 | 791.95 | 7 | KVVYNVA |
| -17.46 | -3.19 | -0.8 | 5.9 | 0 | 839.90 | 7 | YNPSNFV |
| -18.63 | -2.02 | -0.8 | 5.9 | 0 | 853.93 | 7 | YNPSNFL |
